# An overview of the role of platelets in angiogenesis, apoptosis and autophagy in chronic myeloid leukaemia

**DOI:** 10.1186/s12935-017-0460-4

**Published:** 2017-10-30

**Authors:** Lisa Repsold, Roger Pool, Mohammed Karodia, Gregory Tintinger, Annie Margaretha Joubert

**Affiliations:** 1 Department of Physiology, Faculty of Health Sciences, School of Medicine, University of Pretoria, Pretoria, Gauteng South Africa; 2 Department of Haematology, Faculty of Health Sciences, School of Medicine, University of Pretoria, Pretoria, Gauteng South Africa; 3 Department of Internal Medicine, Faculty of Health Sciences, School of Medicine, University of Pretoria, Pretoria, Gauteng South Africa

**Keywords:** Platelets, Chronic myeloid leukaemia, Angiogenesis, Apoptosis, Autophagy

## Abstract

Amongst males, leukaemia is the most common cause of cancer-related death in individuals younger than 40 years of age whereas in female children and adolescents, leukaemia is the most common cause of cancer-related death. Chronic myeloid leukaemia (CML) is a chronic leukaemia of the haematopoietic stem cells affecting mostly adults. The disease results from a translocation of the Philadelphia chromosome in stem cells of the bone marrow. CML patients usually present with mild to moderate anaemia and with decreased, normal, or increased platelet counts. CML represents 0.5% of all new cancer cases in the United States (2016). In 2016, an estimated 1070 people would die of this disease in the United States. Platelets serve as a means for tumours to increase growth and to provide physical- and mechanical support to elude the immune system and to metastasize. Currently there is no literature available on the role that platelets play in CML progression, despite literature reporting the fact that platelet count and size are affected. Resistance to CML treatment with tyrosine kinase inhibitors can be as a result of acquired resistance ensuing from mutations in the tyrosine kinase domains, loss of response or poor tolerance. In CML this resistance has recently become linked to bone marrow (BM) angiogenesis which aids in the growth and survival of leukaemia cells. The discovery of the lungs as a site of haematopoietic progenitors, suggests that CML resistance is not localized to the bone marrow and that the mutations leading to the disease and resistance to treatment may also occur in the haematopoietic progenitors in the lungs. In conclusion, platelets are significantly affected during CML progression and treatment. Investigation into the role that platelets play in CML progression is vital including how treatment affects the cell death mechanisms of platelets.

## Background

Amongst males, leukaemia is the most common cause of cancer-related death in individuals younger than 40 years of age whereas leukaemia is the most common cause of cancer-related death in female children and adolescents [1–3]. One-third of all types of cancer identified in children (1 month to 14 years) are attributed to leukaemia of which 78% are acute lymphoblastic leukaemia (Table 1 and 2) [3].

**Table 1:** Total estimated number of new leukaemia cases in the United States for 2014 [6]

Type	Total	Male	Female
Acute lymphoblastic leukaemia	6020	3140	2880
Chronic lymphocytic leukaemia	15,720	9100	6620
Acute myeloid leukaemia	18,860	11,530	7330
Chronic myeloid leukaemia	5980	3130	2850
Other leukaemia	5800	3200	2600
Total estimated new cases	52,380	30,100	22,280

**Table 2:** Estimated deaths (all age groups) from all types of leukaemia in 2014 in the United States [6]

Type	Total	Male	Female
Acute lymphoblastic leukaemia	1440	810	630
Chronic lymphocytic leukaemia	4600	2800	1800
Acute myeloid leukaemia	10,460	6010	4450
Chronic myeloid leukaemia	810	550	260
Other leukaemia	6780	3870	2910
Total	24,090	14,040	10,050

Leukaemia results from the abnormal formation of white blood cells during the process of haematopoiesis [4]. Leukaemia can be divided into acute- or chronic leukaemia and is further subdivided as either myeloid (from myeloid cells) or lymphoid (from lymphocytes) leukaemia [4].

The most common types of leukaemia are acute myeloid leukaemia (AML), acute lymphoblastic leukaemia (ALL), chronic myeloid leukaemia (CML) and chronic lymphocytic leukaemia (CLL) [4, 5]. Acute leukaemia refers to the rate at which the disease progresses which is in acute cases is rapid development; without treatment the disease would be fatal within a few months of disease onset. The time of disease progression varies according to the type of leukaemia, with accumulation of blood cells that do not mature during haematopoiesis, referred to as blasts [4, 5].

Chronic leukaemia is characterised by a long subclinical period ranging from 3 to 5 years, where there is a delayed build-up of abnormal lymphocytes or myeloid cells. The abnormality differs for each type of leukaemia depending on the genetic mutation present, and results in the lymphocytes or myeloid cells not being able to perform their functions. The latter may not be symptomatic for a prolonged period ranging from months to years [4, 5]. Leukaemia can also arise from erythrocytes or platelets resulting in myeloid leukaemia or from the bone marrow, lymph nodes and spleen [4, 5].

As previously mentioned, the development of the disease is a result of genetic mutation [7]. Genes involved in the regulation of haematopoiesis are commonly mutated in leukaemia, resulting in differentiation defects of haematopoietic cells. Distinctive mutations are implicated in each type of leukaemia [6–8]. Recurrent cytogenetic abnormalities occur in 50% of AML patients and 80% of ALL patients [8]. The rat sarcoma mitogen-activated protein kinase (RAS-MAPK) signalling or phosphatidylinositol 3-kinase (PI3k)/protein kinase B (AKT) signalling allow for proliferation and survival of mutated cells of a haematopoietic origin [7, 8].

Treatment and survival rates of leukaemia depend on the type of genetic mutation responsible and stage at time of diagnosis (which varies per leukaemia type). These include radiation therapy, chemotherapy, targeted therapy and combinations of the three treatments (Table 3) [7].

**Table 3:** Summary of the types of leukaemia including aetiology, genetic markers involved, clinical presentation and treatment [9–11]

Type	Aetiology	Specific markers	Clinical presentation	Treatment
Acute lymphocytic leukaemia	Chromosomal aberration resulting in abnormal transcription factors that affect development of B- and T cells	Hyperdiploidy, Terminal deoxynucleotidyl transferase positive, t(9;22)	Symptoms related to depressed marrow function including anaemia, bone pain and central nervous system manifestations	Chemotherapy, intrathecal therapy, stem cell transplantation
Chronic lymphocytic leukaemia	Chromosomal deletion or possible somatic hypermutation of postgerminal B cells	Trisomy 12, Terminal deoxynucleotidyl transferase negative, t(15;17)	Weight loss, superficial lymph node enlargement and moderate splenomegaly	Drug therapy including chemotherapy, purine analogues and monoclonal antibody therapy, neutrophil growth factors, radiation therapy
Acute myelogenous leukaemia	Oncogenic mutations impede differentiation, accumulating immature myeloid blasts in bone marrow	t(8;21)	Anaemia and bacterial infections	Chemotherapy, stem cell transplantation
Chronic myeloid leukaemia	Tyrosine kinase pathway related to chromosomal translocation of the Philadelphia chromosome	Philadelphia chromosome, t(9;22)	Splenomegaly, hepatomegaly, lymphadenopathy and weight loss	Drug therapy including tyrosine kinase inhibitors, leukapheresis, stem cell transplantation

Stem cell transplantation may also be used as treatment in cases of leukaemia and lymphoma [7]. Stem cell transplantation consists of patients receiving initial high dosages of chemotherapy and/or radiation therapy eliminating the bulk of the patient’s stem cells, the dosage hereof depends on the type of drug administered. Following this therapy, patients receive a transplant of compatible donor stem cells by infusion, replacing the lost stem cells and producing new, unmutated stem cells [7].

Development of innovative targeted-molecular therapy which comprises of drugs that target molecules including those involved in cell growth signalling, tumour blood vessel development and general markers of apoptosis has transformed treatment of leukaemia and specifically CML through the development of tyrosine kinase inhibitors [9–12].

## Chronic myeloid leukaemia

**Fig. 1:**
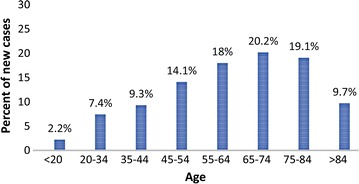
Percent of new chronic myeloid leukaemia cases in 2016 by age group in the United States [13]

**Fig. 2:**
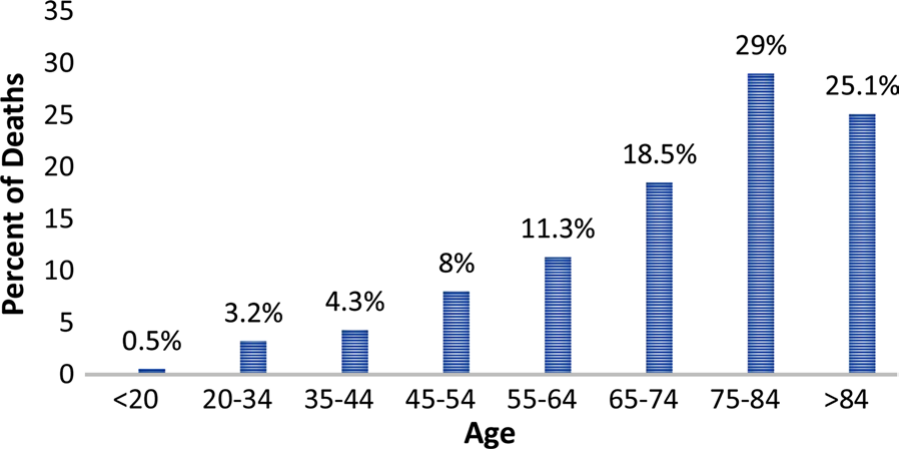
Percent of chronic myeloid leukaemia deaths in 2016 by age group in the United States [13]

CML is a chronic leukaemia of the haematopoietic stem cells affecting mostly adults. In 2016 it was estimated that there would be 8220 new cases of chronic myeloid leukaemia and an estimated 1070 people would die of this disease in the United States (Figs. 1, 2) [13]. Chronic myeloid leukaemia represents 0.5% of all new cancer cases in the United States [13]. CML results from a translocation of the Philadelphia (Ph) chromosome in stem cells of the bone marrow. This, in turn, leads to the collocation of the *Abelson murine leukaemia viral oncogene homolog 1* (*ABL1*) gene from chromosome 9 and the *breakpoint cluster region protein* (*BCR*) gene from chromosome 22 [6, 9–12]. The latter causes the fusion of a *BCR*-*ABL* gene encoding for the aforementioned transcripts and fusion proteins of the BCR-ABL protein including tyrosine-kinase activity involving the phosphorylation of several substrates activating multiple signal-transduction cascades involved in cell proliferation and differentiation [6, 9–12].

Additional genetic events include mutations or deletions of genes namely *p53* and the retinoblastoma protein (*Rb)* following the translocation of the Ph chromosome resulting in the fusion of the *BCR* and *ABL1* genes which allow for the progression of disease [6, 9–12]. The *BCR*-*ABL1* gene may function by hindering apoptosis in targeted stem cells [6]. Inhibition of apoptosis in hematopoietic progenitor cells expressing the fused *BCR*-*ABL* gene is thought to occur through the phosphotyrosine kinase activity of the *BCR*-*ABL* gene. This results in these cells being able to evade dependency on growth factors and resistance to harmful effects of drugs and irradiation [6, 9–12].

CML can be divided into various phases of disease progression; these are the initial chronic stable phase and advanced phase which are partitioned into the earlier accelerated phase and later acute- or blastic phase [6, 9–12]. The chronic phase is usually the phase in which most patients are diagnosed and is characterized by an increased spleen size, while also being able to maintain a normal range of blood counts as established by comparing to reference levels, on therapy with tyrosine kinase inhibitors [6].

Diagnosis of CML includes the presence of splenomegaly, leukocytosis and the incidence of the *BCR*-*ABL1* fusion gene present in leukaemia cells [6, 9–12]. Diagnostic tests include cytogenetic analysis to detect the Ph chromosome, fluorescence in situ hybridization detecting the presence of the mutated *BCR*-*ABL* gene by using fluorescent dyes and the polymerase chain reaction which detects and measures these mutated *BCR*-*ABL* oncogenes [6, 9–12].

Progression of CML from the initial, chronic stable phase to the advanced, accelerated and blastic phases is not well understood. Pathways implicated include the atypical phosphorylation of intracellular proteins such as Crk-like protein (Crkl), mitogen-activated protein kinase 1/2 (Mek 1/2), Rac and c-Jun N-terminal kinase (Jnk) [6, 9–12]. The activation of signal transduction pathways rat sarcoma (RAS) or signal transducer and activator of transcription (STAT) may occur [6, 9–12]. The potential activation of the phosphatidylinositol 3-kinase/AKT pathway that enables apoptosis is also implicated [6, 9–12].

Research conducted to determine the cause of increased bleeding in patients on TKI treatment showed that Dasatinib and, to a lesser degree Imatinib, inhibit platelet function by impairing arachidonic acid- and epinephrine-induced aggregation. The exact mechanism by which this platelet dysfunction is caused is not known; it was shown not to be related to thrombocytopenia or the presence of clonal haematopoiesis [14, 15].

The most widely used treatment for CML is Imatinib, an inhibitor of BCR-ABL tyrosine kinase, a specific inhibitor of the BCR-ABL fusion protein, commonly referred to as a tyrosine kinase inhibitor (TKI) [6, 9–12]. If patients are receptive and responsive to TKI treatment they are likely to survive in excess of 20 years after diagnosis and patients may have an average lifespan of 67 years of age [6, 9–12]. In the case of patients who don’t respond to TKI’s (which is usually about 20% of patients) the disease progresses rapidly in 50% of these patients into the more aggressive acute or blastic phase. In these cases second, third and fourth generation TKI’s are used for treatment as well as haematopoietic stem cell transplants [6, 9–12]. In the other 50% of patients, CML progressively advances to the accelerated phase which may last for months or even years before progressing to the blastic phase [5]. Once the blastic transformation has occurred in patients their survival may only be 3–9 months [6, 9–12].

## Platelets

Platelets are known to serve as a means for tumours to increase growth and provide physical- and mechanical support to elude the immune system and metastasize [16, 17]. There is, however, no literature available on the role that platelets play in CML progression. Due to the fact that platelets fulfil an important role in cancer- and tumour development, their role in CML and potential influence in CML progression are of clinical significance.

Cancer metastasis is directly linked to platelet activity and, in particular, the ability of cancer cells to elude the immune system by formation of platelet-tumour aggregates [18–22]. The latter takes place through the binding of cancer cells (lung-, bone- and breast cancer) to P-selectin and integrins expressed on the membrane of platelets, thus activating the platelets [22].

Binding of cancer cells to platelets via P-selectin consequently results in attraction of platelets to areas of neovascularization and tumour growth by the release of serotonin and thromboxane from platelets [23]. Serotonin is known to have a tumour-stimulatory role and also contributes to cancer-related fatigue, while thromboxane stimulates proliferation and prevents apoptosis of cancer cells [23]. Mitogens including vascular endothelial growth factor (VEGF), platelet derived growth factor (PDGF) and transforming growth factor (TGF) are subsequently released thereby increasing vascularization and growth of the tumour [22–29].

**Fig. 3:**
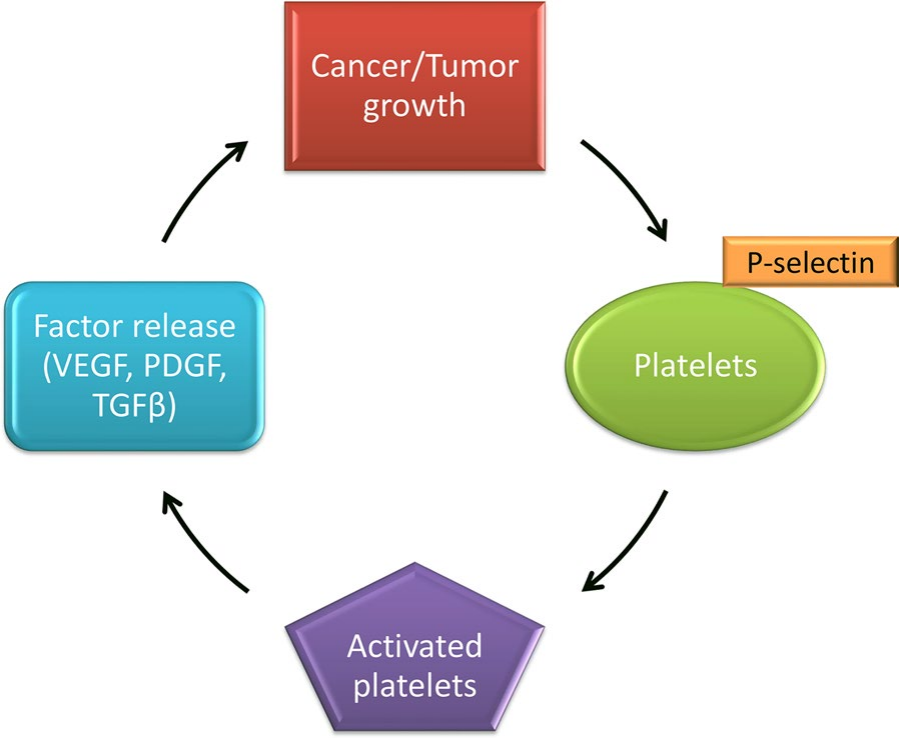
The amplification of tumour growth by binding and subsequent activation of platelets. Cancer cells activate and bind to activated platelets via P-selectin expressed on the platelet membranes. This results in the release of mitogens including VEGF, PDGF and TGFβ that increase growth and vascularization of the tumour mass. These factors further activate platelets following the release of constituents, amplifying the platelet–cancer loop (produced with Microsoft^®^ PowerPoint^®^) [31]

Further activation of platelets ensues from the original tumour; triggering enhanced growth of the tumour as a result of the release of platelets granules [19]. Release of the contents of the granules from platelets hinders the ability of the immune surveillance system against malignancy through cloaking tumour cells and protecting the tumour cells from natural killer (NK) cells by providing a physical barrier and also placing major histocompatibility complex (MHC) class I antigen into the vicinity of the tumour cell surface [8, 30]. This process is referred to as the platelet–cancer loop (Fig. 3) [31].

In a recent publication it was shown that platelets are not solely produced in the bone marrow as conventionally thought, but that platelet biogenesis is predominantly located in the lungs, producing approximately 50% of the total platelets in the circulation [32]. Furthermore, substantial populations of haematopoietic progenitors were found to be produced in the lungs. These progenitors could repopulate the bone marrow in cases of thrombocytopenia and stem cell deficiency [32]. These significant findings demonstrate that there are uncertainties concerning the process of haematopoiesis and specifically how this new source of haematopoiesis may affect our understanding of the aetiology of leukaemia [32].

**Fig. 4:**
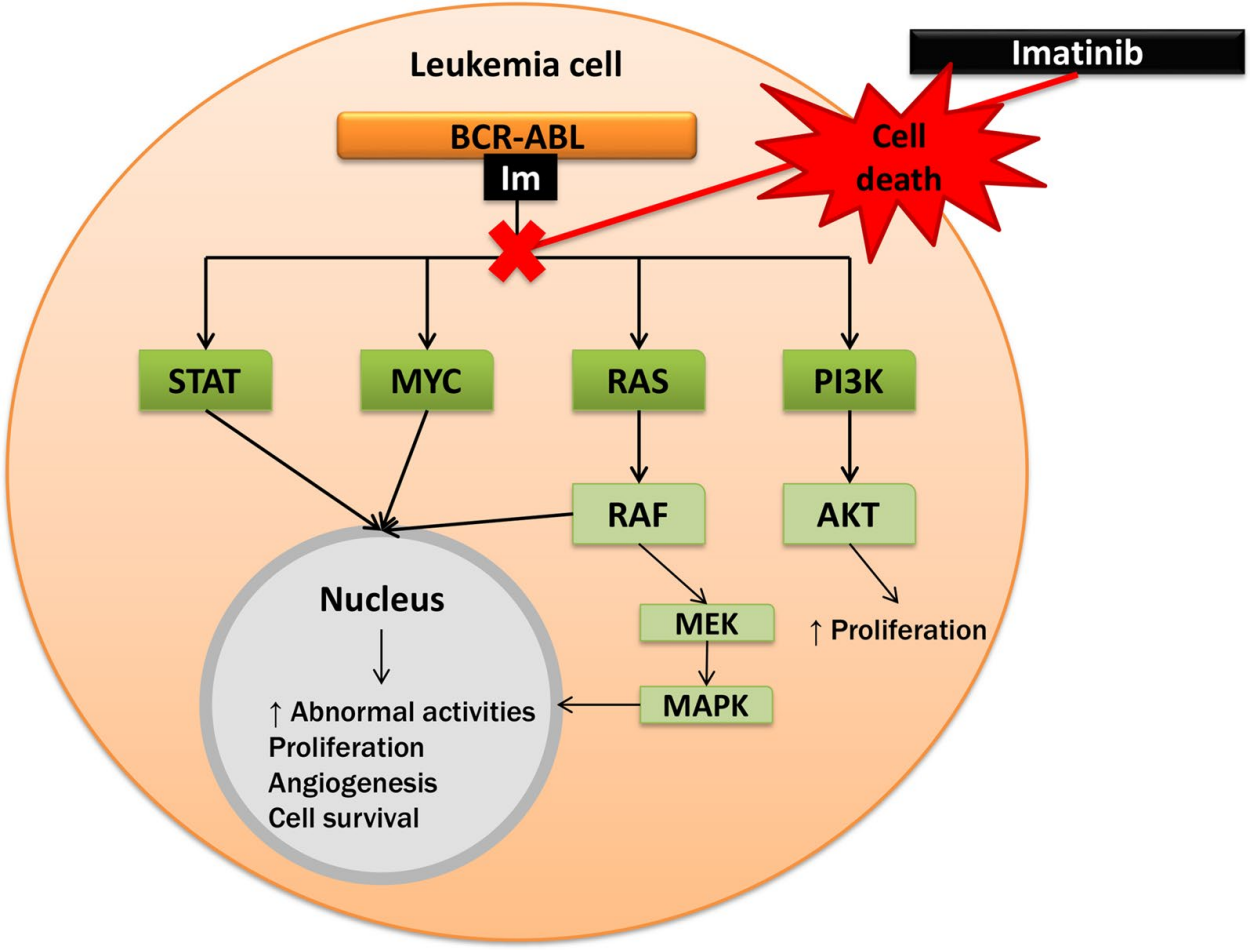
Signal transduction pathway of the *BCR*-*ABL* fusion gene and Imatinib action. Imatinib selectively inhibits the tyrosine kinase activity that is responsible for the signaling pathway illustrated through tyrosine kinase phosphorylation of these interactions. Main signaling pathways associated with the oncogenic activity of the *BCR*-*ABL* gene is MYC, RAS, MAPK, STAT and PI3K. These pathways result in the inhibition of gene transcription, mitochondrial processing of apoptotic reactions and cytoskeletal organization culminating in an increase in abnormal cell activities including unimpeded proliferation, angiogenesis and enhanced cell survival. When Imatinib binds to the tyrosine kinase receptor it in turn deactivates these pathways resulting in cell death of the mutated leukemic cell (produced with Microsoft^®^ PowerPoint^®^) [9–11, 33]

Platelets play an important role in cancer and tumour development, in particular their direct involvement in the process of angiogenesis in tumours. Therapy directed at specifically targeting angiogenesis is a recognized method of treatment, however, it is not well researched in haematological malignancies [33]. The importance of angiogenesis-targeted therapies in CML has recently become clear as the occurrence of TKI resistance and specifically Imatinib resistance increases (Fig. 4).

Failure of patients to respond to Imatinib treatment can be a result of acquired resistance ensuing from mutations in the BCR-ABL 1 tyrosine kinase domain, loss of response and poor tolerance [33]. In CML, this resistance has recently become linked to bone marrow (BM) angiogenesis which aids in the growth and survival of leukaemia cells [33]. However, with the discovery of the lungs as a site of haematopoietic progenitors, this may indicate that CML resistance is not localized to the bone marrow and that the mutations leading to the disease and resistance to treatment may also occur in the haematopoietic progenitors in the lungs [32].

## Angiogenesis

Angiogenesis is a well-known contributor to cancer progression and is defined as a closely-controlled biological process which takes place during foetal development of blood vessels and wound healing [34]. Angiogenesis is a process associated with the formation of new vascular sections onto a pre-existing vascular system [35, 36].

Tumour angiogenesis, the process leading to the formation of new blood vessels within the tumour mass, provides cancer cells with oxygen and nutrition and plays a central role in cancer cell survival [35, 36]. It also promotes tumour growth and possible development of distant metastases [34–36].

The angiogenesis-related proteins released during angiogenesis can be differentiated into the angiogenic activators and the angiogenic inhibitors [30, 37]. Angiogenesis-activating proteins include VEGF, PDGF and matrix metallopeptidase-9 (MMP-9), while the angiogenic inhibitors include transforming growth factor β (TGFβ) [30, 37, 38]. These angiogenesis-regulatory factors are released from activated platelets in circulating blood of patients with cancer or the development of tumours [38–40].

VEGF is a dimeric glycoprotein and a member of the PDGF family which contributes to angiogenesis by promoting endothelial cell growth, maturation and survival, enhancing vascular permeability and inhibiting apoptosis [41–44]. A wide variety of human tissues express low levels of VEGF (around 108 pg/ml) [41–44]. High levels (around 238 pg/ml) are produced where angiogenesis is required such as in foetal tissue, the placenta, the corpus luteum, as well as in the vast majority of human tumours including breast, colorectal, bladder and ovarian cancers [45]. Studies have shown that prostate- and colorectal cancer patients have increased serum VEGF levels when compared to healthy individuals [36, 46, 47].

It was reported that TGFβ, another angiogenic-regulating factor released by platelets, also plays a role in the inhibition of the antitumour activity of T-cells, NK cells, neutrophils, monocytes and macrophages involved in regulating cancer progression [48].

PDGF is present in a number of cells including platelets, fibroblasts, keratinocytes, myoblasts, astrocytes, epithelial cells and macrophages [45]. Expression of PDGF and platelet derived growth factor receptors (PDGFRs) are dynamic and characterized by a constant change in levels; their biosynthesis and processing are controlled at various levels where increased expression or levels are indicative of several diseases and pathological conditions which are categorized into three causative disease groups namely tumours, vascular diseases and fibrosis [49].

Levels of PDGF, however, can be upregulated by a variety of stimuli including hypoxia, thrombin, cytokines and growth factors such as TGFβ [49]. Studies have shown increased PDGF signalling in epithelial types of cancer which affected tumour growth, angiogenesis, invasion and metastasis [49]. This may be explained by the fact that both PDGFR-α and PDGFR-β engage in signaling pathways namely RAS-MAPK and PI3K known to be involved in cellular- and developmental responses including stimulation of cell growth, differentiation and migration [49].

**Fig. 5:**
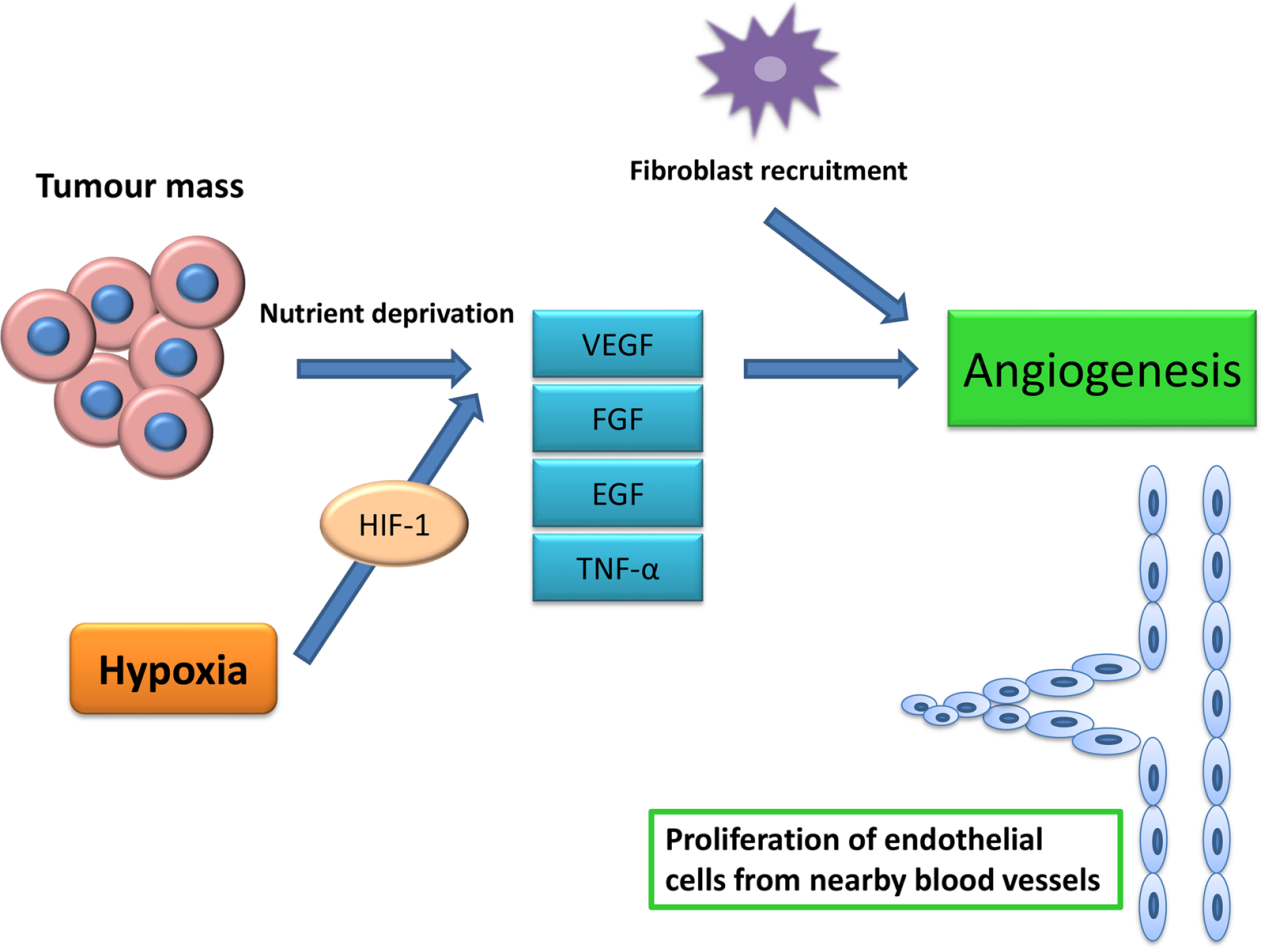
Angiogenesis in cancer. Nutrient deprivation and hypoxia signal the necessity of oxygen and nutrients to the tumour mass and thus activate the process of angiogenesis through increased expression of proangiogenic genes including via HIF-1. This includes VEGF which results in vasodilation of pre-existing blood vessels via generation of nitric oxide, EGF, Ang1 and bFGF which stimulates proliferation, migration and assembly of the endothelium. Integrins α_v_β_3_ and α_5_ mediate cell migration and spreading and PDGF recruits smooth muscle cells for the formation of a new basement membrane of forming vessels (produced with Microsoft^®^ PowerPoint^®^) [34]

The process of angiogenesis (Fig. 5) is thought to be primarily caused by hypoxia in tumours which activate hypoxia-inducible factor-1 (HIF-1) [35]. HIF-1 is responsible for increased expression of pro-angiogenic genes including VEGF [34]. VEGF mediates the process of angiogenesis through vasodilation of pre-existing blood vessels via generation of nitric oxide [34]. VEGF is a main contributor to angiogenesis by promoting endothelial cell growth, maturation and survival, enhances vascular permeability and inhibits apoptosis [36, 43, 44].

Nutrient deprivation within a tumour mass also signals the release of various angiogenic molecules [16, 17, 45–50]. The release of VEGF, epidermal growth factor (EGF), angiopoietin 1 (Ang1) and basic fibroblastic growth factor (bFGF) stimulates proliferation, migration and assembly of the endothelium, while integrins α_v_β_3_ and α_5_ mediate cell migration and spread [34–36]. Formation of a new basement membrane is essential in maturation of newly formed vessels which takes place through recruitment of smooth muscle cells via PDGF [41].

Most of the above-mentioned angiogenesis-regulatory factors are released specifically from the α-granules of platelets, which also play a role in vascular repair and cell to cell interactions [37]. Platelets contain 3 types of secretory granules including α-granules, dense granules and lysosomes. It has, however, been shown that platelets can release either pro-angiogenic factors or anti-angiogenic factors differentially in response to various tissue stimuli [37]. This suggests that platelets may hold clinical implications once the mechanism of differentiating release of pro- and anti-angiogenic factors is elucidated to target specific release of antiangiogenic factors at tumour sites [37].

## Cell death: apoptosis

**Fig. 6:**
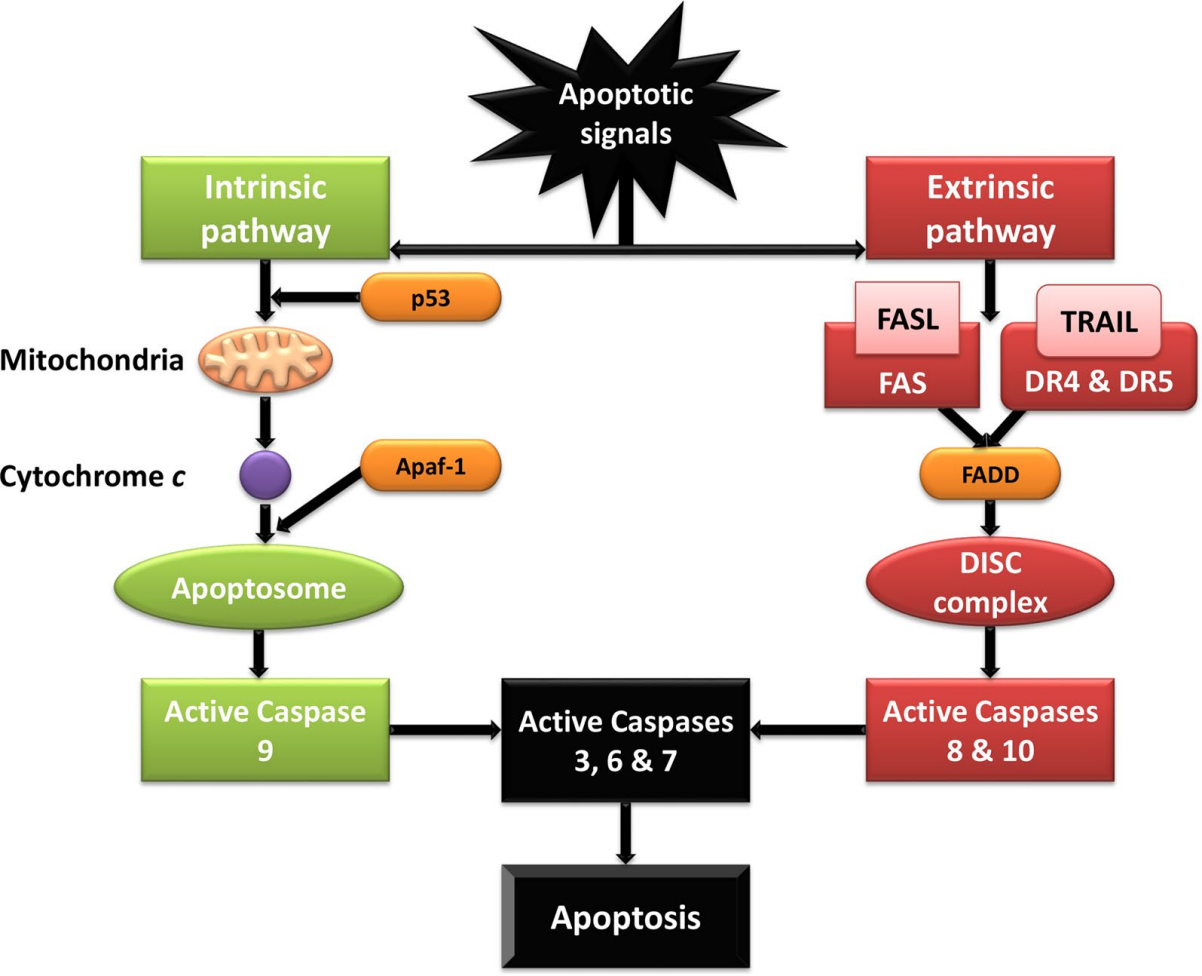
Graphical representation of the intrinsic and extrinsic apoptotic pathway. The intrinsic apoptotic pathway is represented in green, indicating the release of cytochrome *c* into the cytoplasm from the mitochondria following apoptotic signals to the cell. Following its release, cytochrome *c* binds to apoptotic protease activating factor 1 (Apaf-1) forming the apoptosome which in turn recruits procaspase 9. Procaspase 9 binds to the apoptosome activating caspase 9 which sequentially activates the effector caspases 3, 6 and 7 resulting in the execution phase of apoptosis. The extrinsic apoptotic pathway is shown in red and depicts the interaction of DRs with their corresponding death ligands following death signals to the cell. The binding of the death ligands to their DRs results in the release of adaptor molecules such a Fas-associated death domain (FADD) which employs inactive procaspases 8 and 10, forming the DISC and subsequent activation of the effector caspases resulting in apoptosis (produced with Microsoft^®^ PowerPoint^®^) [54–58]

Many endogenous angiogenesis inhibitors have been shown to induce apoptosis in vivo [51]. Apoptosis is characterised by membrane blebbing, cell shrinkage, hypercondensation of chromatin and formation of apoptotic bodies, activated by either the intrinsic and/or the extrinsic pathways [52, 53]. Both of these pathways include the interaction of death receptors with death ligands and activation of caspases (Fig. 6). These can be divided into two classes namely the initiator caspases (caspase 8 or 9) and the executioner caspases (caspase 3, 6, and 7) [54, 55].

In nucleated cells, the intrinsic apoptosis pathway is initiated by stimuli which trigger cytochrome *c* to be released from the mitochondria, and, in turn recruit’s initiator caspase 9, thereby activating executioner caspase 3 resulting in apoptosis [56]. During the extrinsic apoptotic pathway in nucleated cells, death receptors (DRs) including DR5 bind to death ligands which employ the initiator caspases 8 and 9, forming the death-inducing signalling complex (DISC) and activation of the effector caspase 3, ensuing in apoptosis [52, 57, 58].

The removal of apoptotic cells is a result of phosphatidylserine (PS) collecting on the external layer of the cell membrane which is initiated by activation of the calcium-dependent phospholipid scramblase and signals macrophages to stimulate the removal of the apoptotic cells [56]. Once the PS has been externalized, a distinct characteristic of apoptosis, it is possible to quantify the extent of the PS-flip as binding sites are revealed during the flip [59].

Apoptosis is closely associated with occurrences within the nucleus and is consequently questioned in platelets since they lack this cellular component [60, 61]. Platelets display characteristic signs of nucleated apoptosis including membrane blebbing, loss of the integrity of the platelet membrane and microparticle release [60, 61]. The ability of platelets to undergo apoptosis is a result of mitochondrial presence which contributes to mitochondrial deoxyribonucleic acid (DNA) and messenger ribonucleic acid (mRNA). Mitochondrial DNA and mRNA aid in the platelets’ ability to synthesise proteins contained within platelet granules [62–64].

Thus, even though platelets do not possess a nucleus, they exhibit biological apoptotic signals during stressed conditions including activation of caspase 3 and exposure/externalisation of phosphatidylserine [65, 66]. Kile [67] showed that platelets do undergo apoptosis via the intrinsic apoptotic pathway that also regulates the platelets’ lifespan.

The intrinsic apoptotic pathway in platelets, comparable to the process in nucleated cells, is characterised by activation of Bak and Bax, members of the B-cell lymphoma 2 (Bcl-2) protein family which promote apoptosis, triggering damage of the mitochondria and releasing cytochrome *c* and other apoptotic proteins from the mitochondrial intermembrane space. The release of cytochrome *c* allows for the formation of the Apaf-1 apoptosome and subsequent recruitment of initiator procaspase 9. Binding to the apoptosome activates caspase 9 and leads to the activation of effector caspase 3, culminating in the execution phase of apoptosis [58]. Upstream of caspase 3 activation and PS exposure, the mitochondrial inner transmembrane potential is depolarized in platelets, similar to the mechanism of nucleate cellular apoptosis (which is the programmed process of apoptosis in nucleated cells) [64, 68].

The resulting externalisation of PS then allows for removal of apoptotic platelets. In platelets, PS is also expressed on the cell surface, however, it can only be recognized by macrophages for phagocytosis by recognition via human cluster of differentiation 36 (CD36) present on the membrane of human platelets [65–69]. The externalisation of PS in platelets seems to also occur independently of the intrinsic apoptotic pathway playing an important role in formation of thrombin by assembling the pro-thrombinase complex [66–68].

## Cell death: autophagy

**Fig. 7:**
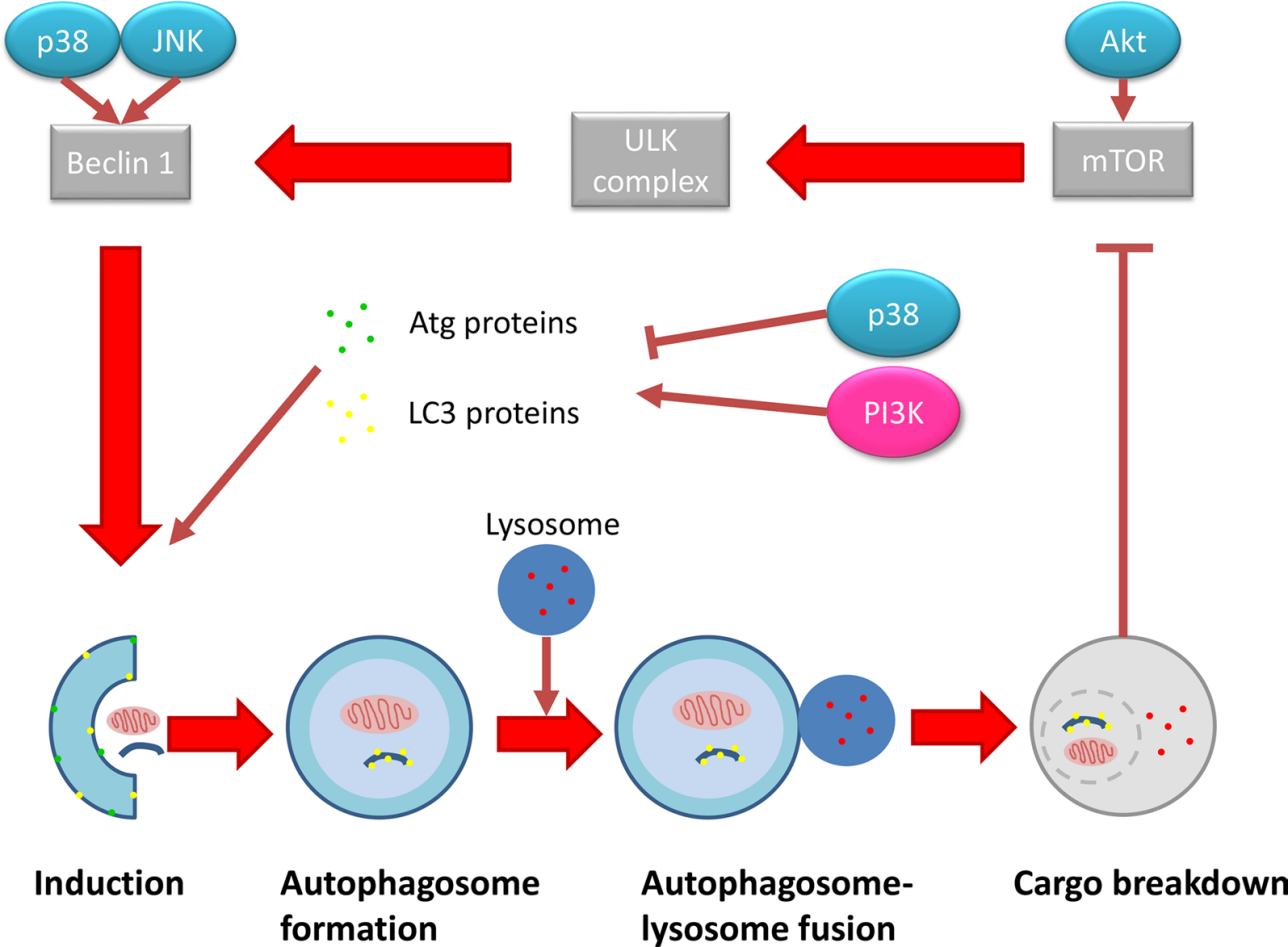
Overview of autophagy in cells. Autophagy is activated through starvation signals stimulating Akt and thus inactivating mammalian target of rapamycin (mTOR) by detaching it from the uncoordinated 51-like kinase (ULK) complex which in turn activates the ULK complex. Beclin-1 is subsequently activated recruiting Atg proteins necessary for autophagosome formation. These Atg proteins aid in employing and converting LC3-I to LC3-II by conjugation to phosphatidylethanolamine (PE) and Atg3 and 7. Upon the formation of the autophagosome, it fuses with lysosomes forming the autolysosome, wherein breakdown of the cargo takes place to recycle amino acids and fatty acid for further energy generation (produced with Microsoft^®^ PowerPoint^®^) [69, 70]

In addition to apoptosis in platelets, the role of autophagy and the biological markers, including autophagy-related proteins (Atg) and quantification of the conversion of light chain 3-I (LC3-I) to LC3-II have not been researched extensively in platelets. Since platelets do contain small amounts of functional mitochondria, it has been proposed to share characteristics of nucleated autophagy mechanisms and markers (Fig. 7) [66]. Autophagy’s ability to maintain cellular homeostasis and adjustment to starvation is of importance in platelets as their lifespan is only about 10 days in humans [69, 70]. Autophagy can also be triggered continuously under certain stressed conditions such as starvation, cellular injury and contact with certain chemicals such as lithium, which leads the cell to progressively degrade vital cytoplasmic components, essentially digesting itself [69, 70].

The occurrence of autophagy in platelets is essential in maintaining homeostasis within platelets and in the number of platelet populations [71]. The incidence of autophagy is not well documented in platelets. Literature has shown that platelets do express Atg proteins and the process is also activated by the inhibition of mTOR [71, 72]. A defect in platelet autophagy may result in compromised platelet adhesion and aggregation impacting on coagulation and the resulting formation of a platelet plug during damage to blood vessels [71].

## Conclusion

CML patients have abnormal megakaryocytes that can deliver unusual blast fragments to the peripheral blood and patients are frequently found to have large and heterogeneous platelets. Additionally, TKI treatment has been shown to induce platelet dysfunction and may result in coagulation abnormalities and an increased incidence of bleeding [73–76]. Since platelets are significantly affected during CML progression and treatment, investigation into the role that platelets play in CML progression is of importance, including how treatment effects the cell death mechanisms of platelets. In light of new research implicating the lungs as an additional production site not only for platelets, but also haematopoietic progenitors, research into platelet involvement in CML is of critical importance.

## Abbreviations

ABL1: Abelson murine leukaemia viral oncogene homolog 1
AKT: protein kinase B
ALL: acute lymphoblastic leukaemia
AML: acute myeloid leukaemia
Ang1: angiopoietin 1
Apaf-1: apoptotic protease activating factor 1
Atg: autophagy-related genes
Bcl-2: B cell lymphoma 2
BCR: breakpoint cluster region protein
bFGF: basic fibroblastic growth factor
BM: bone marrow
CD: cluster of differentiation
CLL: chronic lymphocytic leukaemia
CML: chronic myeloid leukaemia
Crkl: Crk-like protein
DISC: death-inducing signaling complex
DNA: deoxyribonucleic acid
DRs: death receptors
EGF: epidermal growth factor
FADD: Fas-associated death domain
HIF-1: hypoxia-inducible factor-1
Jnk: c-Jun N-terminal kinase
LC3: light chain-3
MAPK: mitogen activated protein kinases
Mek 1/2: mitogen-activated protein kinase 1/2
MHC: major histocompatibility complex
MMP-9: matrix metallopeptidase-9
mRNA: messenger ribonucleic acid
mTOR: mammalian target of rapamycin
NK: natural killer
PDGF: platelet derived growth factor
PDGFRs: platelet derived growth factor receptors
PE: phosphatidylethanolamine
Ph: Philadelphia
PI3K: phosphatidylinositol 3-kinase
PS: phosphatidylserine
RAS: rat sarcoma
RAS-MAPK: rat sarcoma mitogen-activated protein kinase
STAT: signal transducer and activator of transcription
TGFβ: transforming growth factor β
TKI: tyrosine kinase inhibitor
ULK: uncoordinated 51-like kinase
VEGF: vascular endothelial growth factor
